# Regulatory Mechanism of Copper Oxide Nanoparticles on Uptake of Different Species of Arsenic in Rice

**DOI:** 10.3390/nano11092228

**Published:** 2021-08-29

**Authors:** Qianhua Wu, Jiyan Shi, Xiaohan Jiang, Hanxin Wu

**Affiliations:** 1 Department of Environmental Engineering, College of Environmental and Resource Sciences, Zhejiang University, Hangzhou 310058, China; wqhwqh@zju.edu.cn (Q.W.); 11814047@zju.edu.cn (X.J.); wuhanxin@zju.edu.cn (H.W.); 2 Key Laboratory for Water Pollution Control and Environmental Safety, Zhejiang University, Hangzhou 310058, China

**Keywords:** copper oxide nanoparticles, arsenic, accumulation, gene

## Abstract

Copper oxide nanoparticles (CuO NPs) are widely used as a fungicide in agriculture. The application of CuO NPs in agriculture affects the growth of rice and metal accumulation in rice. However, the mechanism of CuO NPs on arsenic (As) accumulation in rice remains unclear. In this study, a hydroponic culture was produced to investigate the mechanism of the effect of 50 and 100 mg L^−1^ CuO NPs on As accumulation in rice. Our results showed that CuO NPs decreased As(III/V) accumulation in the roots and shoots by adsorbing As(III/V), oxidizing of As(III) on the surface, and thickening the root cell wall. The addition of CuO NPs regulated the expression of the *OsNIP1;1*, *OsHAC1;1,* and *OsHAC4* genes, which decreased As(III) transport and promoted As(V) reduction in the roots. Moreover, when CuO NPs were co-exposed to As, a negative correlation between the concentration of Cu and As in rice was also found in our study. However, CuO NPs significantly increased Cu accumulation in rice and constrained the rice growth. In conclusion, CuO NPs might be a promising way to decrease As accumulation in rice, but the negative effects such as growth inhibition should be further considered. Therefore, the application of CuO NPs in rice plants should take a more restrained approach.

## 1. Introduction

Nanomaterials refer to the materials composed of 1 to 100 nanometer scale materials. Nanoparticles are characterized by a small volume, large surface area, excellent chemical reaction performance, and stronger oxidation ability. Nanomaterials are used in many fields because of the remarkable enhancement of optics, mechanics, electricity, structure, and magnetism of nanomaterials [1]. Copper oxide nanoparticles (CuO NPs) are widely used as a fungicide in agriculture because of their destructive effect on bacteria and fungi at both the cellular level and the molecular level [2,3]. In addition, CuO NPs are used as a plant growth promoter in agriculture when soil is infected by pathogenic bacteria [4,5]. CuO NPs also increase the uptake of nutrient elements by plants [6].

CuO NPs not only affect plant nutrient uptake, but also affect plant Arsenic (As) uptake. Recent studies have shown that CuO NPs could reduce the content of As in rice tissues and the accumulation of As(III) in rice grains [7,8]. Arsenic is a toxic metalloid and can enter the human body through the food chain. Therefore, the application of CuO NPs may be a new and promising remediation method to reduce As accumulation in rice [7]. However, previous studies have shown that over 500 mg kg^−1^ of CuO NPs could inhibit rice growth and destroy the cell structure [9,10]. Moreover, CuO NPs also disrupt the cell cycle and have a toxic effect on cell division, thereby inhibiting root growth [9].

The toxicity mechanism of different species of As that are taken up by rice has been well studied. As(III) is actively transported through the plasma membrane of aquaporin NIPs in rice, whereas As(V) is transported by a phosphate transporter (PHT1) [11,12,13]. It is well known that inorganic As(III) is more toxic to plants than inorganic As(V), and As(III) efflux is an important mechanism of As detoxification in plants [14]. Additionally, the detoxification mechanism of As(V) in rice roots reduces As(V) to As(III) and also promotes As(III) efflux [15]. Transporter genes of *OsPT1*, *OsPT4*, and *OsPT8* have been reported to be involved in the uptake and transport of As(V) in rice [16,17]. Genes of *OsHAC1;1*, *OsHAC2;1*, and *OsHAC4* mediate the reduction of As(V) in rice roots [18,19]. The genes *OsLsi1* and *OsLsi2* are located in the extracellular and endothelial layers, which have been confirmed to be involved in the influx and efflux of As(III) in rice [11]. A recent study found that *OsFTIP7* is involved in responses and increases tolerance by regulating the auxin biosynthesis in rice after a high dose of CuO NPs exposure [20]. Therefore, CuO NPs might also regulate As accumulation in rice by regulating As related genes.

In a previous study, it was shown that CuO NPs could remove As in a solution through adsorption, and with a higher concentration of CuO NPs, removal efficiency is higher [21]. Therefore, CuO NPs could decrease As accumulation in rice by adsorbing As. However, the mechanisms of CuO NPs adsorption and its regulation of rice gene expression, which subsequently affects different species of As that accumulate in rice, remains unclear. This study aimed to explore the effect of CuO NPs on rice uptake and the accumulation of As(III/V) at the cell level and at the molecular level. These findings would demonstrate the mechanism of CuO NPs on different species of As uptake and accumulation in rice and would also bring insight into the application of CuO NPs in agriculture.

## 2. Materials and Methods

### 2.1. Chemicals and Materials

Copper oxide nanoparticles (purity ≥ 99.8%) were purchased from Beijing NaChen Technology Co., Ltd. (Beijing, China). A small amount of CuO NPs and 2 mL absolute ethyl alcohol were placed in a centrifuge tube and were ultrasonically dispersed for 1 min and then dried in a nickel mesh. The morphology and particle size of CuO NPs were observed using a transmission electron microscope (TEM, Hitachi H-7650, Tokyo, Japan). A small amount of the CuO NPs was placed on the aluminum stage, and the elemental composition was analyzed using an energy dispersive spectrometer (EDS). The morphology, particle size, and elemental composition of the CuO NPs are shown in Figure S1 of the Supplementary Materials.

### 2.2. Rice Cultivation and Experiment Setup

The rice cultivar *Huanghuazhan* was chosen as our study material. Rice seeds were sterilized in 30% H_2_O_2_ solution for 15 min and were then rinsed with deionized water three times. These seeds were placed in phytotron and were germinated in the dark at 26 °C. After 1 week, the rice seedlings were transplanted and cultured in 1/2 strength nutrient solution for 1 week. Then, the seedlings were transplanted and cultured in complete nutrient solution for another 1 week. The complete nutrient solution contained 2.85 mM NH_4_Cl, 0.32 mM NaH_2_PO_4_, 1 mM K_2_SO_4_, 1 mM CaCl_2_, 1.7 mM MgSO_4∙_7H_2_O, 35 μM FeCl_3_∙6H_2_O, 18 μM H_3_BO_4_, 0.52 μM (NH_4_)_6_∙Mo_7_O_24_∙4H_2_O, 9 μM MnCl_2_∙4H_2_O, 0.15 μM ZnSO_4_∙7H_2_O, 0.15 μM CuSO_4_∙5H_2_O, and 70 μM citric acid monohydrate. The 21-day-old seedling was rinsed with deionized water, and every two seedlings were transplanted into a 1 L PVC black bottle containing 1 L nutrient solution. The culture system of the rice seedlings is shown in Figure S2. Sodium arsenite (NaAsO_2_) and sodium arsenate (Na_2_HAsO_4_·7H_2_O) were used as the source of As(III) and As(V). Three levels of As(III/V) (0, 0.5 and 1 mg L^−1^) and CuO NPs (0, 50 and 100 mg L^−1^) were selected in this experiment. Seedlings without As and CuO NP exposure were part of the control group (CK). Seedlings exposed to As(III) and As(V) were Group A and B, respectively. There were 13 treatments in this study: CK, 0.5A, 1A, 0.5B, 1B, 0.5A+50, 0.5A+100, 1A+50, 1A+100, 0.5B+50, 0.5B+100, 1B+50, and 1B+100. Each treatment conducted in 3 replicates. The seedlings were cultured in the phytotron at 26 °C, 70% humidity, and 10,000 Lx, 16 h light/8 h dark. The nutrient solution was renewed every 3 days to maintain the species of As exposed to the rice [22,23]. After 14 days of exposure, the rice seedlings were harvested, and the shoot and root biomasses were recorded. The roots and shoots of the rice were rinsed with tap water and were deionized water three times. Fresh roots with the same position were completely immersed in 2.5% glutaraldehyde solution in order to observe the cell morphology. The remaining roots and shoots were frozen with liquid nitrogen and were stored in the dark at −80 °C until analysis.

### 2.3. Elements Analysis

The root and shoot samples were dried in a vacuum freeze dryer for 48 h. Then, the samples were ground into a powder. About 0.2 g of the samples were weighed in a polytetrafluoroethylene digestion tube, and 10 mL of HNO_3_ was added to the samples and left overnight. The digi block method was used for digestion [24]. In brief, the samples were heated to 100 °C for 2 h until the solution in the tube was clear. Then, the solution was transferred to a 25 mL volumetric flask and was filtered through a 0.22 μm filter. The concentrations of As and Cu in the rice roots and shoots were determined by inductively coupled plasma mass spectrometry (ICP-MS, PlasmaQuant, analytikjena, Jena, Germany). The methanol extraction method was used to extract different species of As in the roots and shoots [25]. About 0.2 g of fresh roots and shoots were weighed in an agate mortar and were ground into powder using liquid nitrogen. Then, the powder was transferred to a centrifuge tube via 10 mL 50% methanol solution and underwent ultrasonic processing for 30 min. Centrifugation was performed at 4 °C for 15 min at 5000 r/min, and the supernatant was obtained. The extraction was repeated once. Then, 10 mL deionized water instead of 50% methanol to was for extraction once. After three extractions, all of the supernatant was mixed. The different species of As including As(III), dimethyl arsenic [DMA(V)], methyl arsenic [MMA(V)], and As(V) in the samples were determined by HPLC-ICP-MS (Agilent infinity 1260 II, HPLC Agilent Technologies 7800 ICP-MS, Santa Clara, CA, USA).

### 2.4. The Observation of CuO NPs and Rice Roots

CuO NPs and nutrient solutions were removed to a 10 mL centrifuge tube and were then freeze-dried. The species of As and Cu on the surface of the CuO NPs were analyzed by means of X-ray photoelectron spectroscopy (XPS, Thermo Scientific K-Alpha, Waltham, MA, USA) in our study.

Nine treatments (CK, 0.5A, 1A, 0.5A+50, 1A+100, 0.5B, 1B, 0.5B+50 and 1B+100) were selected for TEM-EDS observation. The fresh roots were immersed in 2.5% glutaraldehyde, rinsed with 0.1 M phosphate buffer, and then fixed with 1% osmic acid solution. Then, the samples were rinsed with 0.1 M phosphate buffer again and were dehydrated by ethyl alcohol and acetone. All of the samples were treated with a mixture of Spurr embedding agent and acetone. Then, the samples were sliced by using an ultra microtome (EM UC7, Leica, Weztlar, Germany) and were stained with a lead citrate solution and a uranium oxyacetate 50% ethanol saturated solution for 5~10 min. The samples were placed on nickel mesh and were observed with a transmission electron microscope (TEM), and the composition was determined using an energy dispersive spectrometer (EDS).

### 2.5. Real-Time Quantitative PCR

According our results regarding the As concentration in the rice roots, 50 or 100 mg kg^−1^ CuO NPs co-exposed with same level of As showed no differences in the total As observed in the roots. Therefore, five treatments (CK, 1A, 1A+100, 1B, 1B+50) that showed higher rice root biomass were selected to perform the qPCR process. The Plant RNA Extraction Kit v1.5 (BioFit, Shanghai, China) was used, and qPCR was performed by means of the Allwegene Tech. Co., Ltd. (Shanghai, China). First, 25~100 mg of the root system was cut and placed into a 10 mL centrifuge tube, and the plant RNA Extraction Kit v1.5 (BioFit, Shanghai, China) was used to extract the RNA. Solution A was added and was homogenized for 20 s using a homogenizer. A total of 1 mL of homogenate was transferred to a 1.5 mL centrifuge tube. Additionally, 300 µL of Solution B and 200 of µL chloroform were added and then oscillated for 30 s and fully emulsified. RNA samples were obtained after several times of extraction and centrifugation.

Reverse transcription and amplification were performed using the Goldenstar RT6 cDNA Synthesis Kit Ver 2. Reverse transcription system: 0.2 µL of RNA template, 1 µL of gDNA remover, 1 µL of 10XgDNA remover buffer. An amount of 10 µL RNase-free water was added to the total volume of the system. Samples were incubated at 42 °C for 2 min and then incubated at 60 °C for 5 min. Samples were cooled quickly on ice. Then, 1 µL dNTP Mix, 1 µL Randomer primer, 4 µL 5 × GoldenstarTM Buffer, 1 µL DTT(2M), 1 µL Goldenstartm RT6 enzyme, and 2 µL RNase-free water were added. The cDNA products obtained by reverse transcription were diluted appropriately as a qPCR template, and 2×T5 fast qPCR mix (SYBR Green I) amplified the target gene. The qRT-PCR program was set as follows: 95 °C, 1 min; 95 °C, (15 s; 60 °C, 15 s; 72 °C, 30 s) 40 cycles; 50 °C to 95 °C for melting curve detection. A total of 11 genes involved in As transport and transformation were evaluated. The primers used are shown in Table S1. According to the Ct value, the relative quantification was calculated using the 2^(−ΔΔCt)^ formula. Actin was used as a reference gene in the qPCR reaction. The CK treatment was used as the control sample for calibration.

### 2.6. Data Analysis

All the of the data are means of triplicate ± standard deviation. The least significant difference (Tukey’s Test) between the different treatments was measured at *p* < 0.05. All data were statistically analyzed using IBM SPSS Statistics software (version 19.0, Armonk, NY, USA). Diagrams were plotted by Origin Lab (version 8.5, Northampton, MA, USA).

## 3. Results

### 3.1. Adsorption of As by CuO NPs

**Figure 1:**
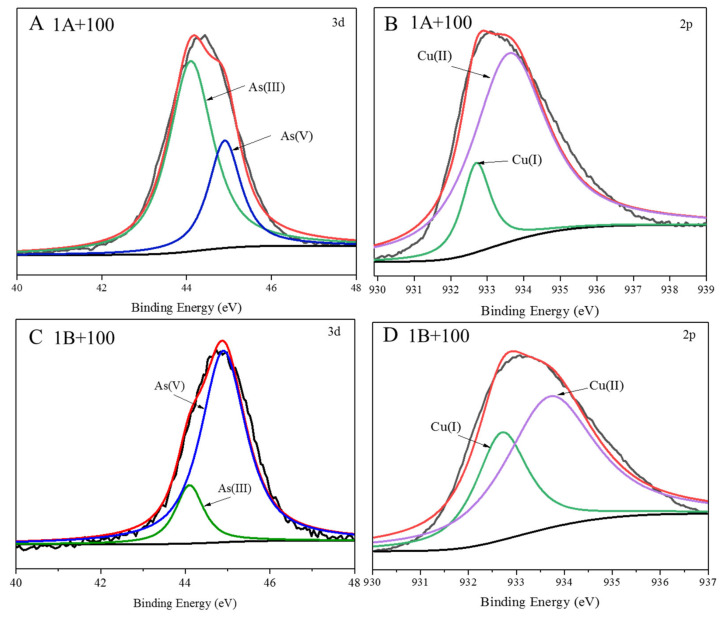
Species of As and Cu on the surface of CuO NPs as determined by XPS. Spectra of CuO NPs with As(III) exposure (**A**,**B**) and As(V) exposure (**C**,**D**). The green and blue line in A and C images were represented As(III) and As(V) respectively; the green and purple line in B and D images were represented Cu(I) and Cu(II) respectively.

The species of As and Cu on the surface of the CuO NPs are showed in Figure 1. The NIST X-ray photoelectron spectroscopy database (version 4.1, Gaithersburg, MD, USA) issued by the National Institute of Standards and Technology showed that the binding energies of As(III) and As(V) were 44.1 eV and 45.7 eV, respectively, and the binding energies of Cu(I) and Cu(II) were 932.6 eV and 937.8 eV, respectively. Our results showed that As(III/V) was adsorbed on the surface of CuO NPs. It is worth noting that there was a small amount of As(V) on the surface of CuO NPs when the rice was exposed to As(III) (Figure 1A). There was also a small amount of As(III) on the surface of CuO NPs when rice was exposed to As(V) (Figure 1C). Meanwhile, Cu(I) also was detected on the surface of CuO NPs under the 1A+100 and 1B+100 treatments (Figure 1B,D). These results showed that adsorption and species transformation of As occurred on the surface of CuO NPs.

### 3.2. Accumulation and Speciation of As in Rice

**Figure 2:**
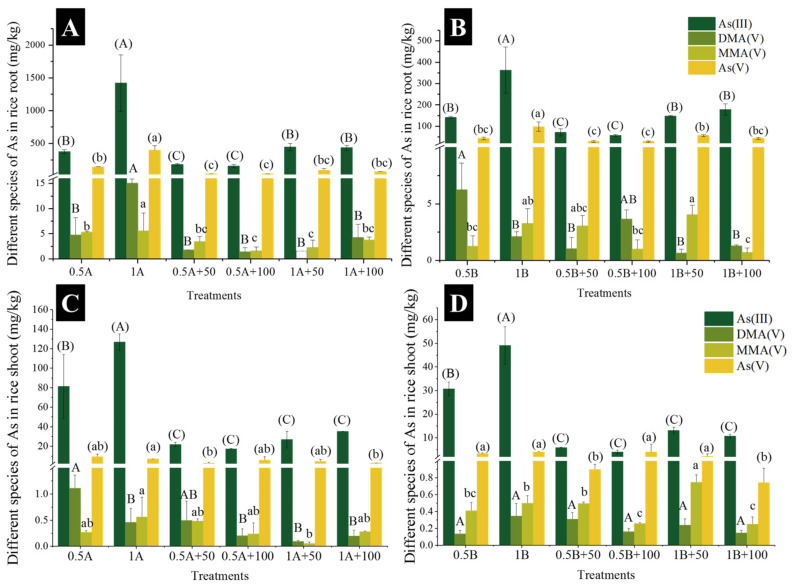
The concentrations of different species As in rice roots (**A**,**C**) and shoots (**B**,**D**). Different lowercase letters indicate significant differences between different treatments (*p* < 0.05).

The accumulation and speciation of the As in the roots and shoots are shown in Figure 2. The total As concentrations in the roots and shoots increased with the exposure of As concentrations. In addition, the accumulation of As in rice under As(III) treatment was higher than that under As(V) treatment when exposed to same level of As(III/V). The application of CuO NPs significantly decreased As concentrations in the roots and shoots. Notably, 50 or 100 mg kg^−1^ CuO NPs co-exposed to the same level of As showed no differences on the total of As in the roots (Figure 2A).

The speciation of the As in the rice was further analyzed, and As(III) and As(V) were dominant in the rice (Figure 2, Figure S3). Compared to 0.5 or 1 mg L^−1^ As(III) exposure alone, the concentrations of As(III) and As(V) in the rice roots were significantly decreased with the addition of 50 and 100 mg L^−1^ CuO NPs (Figure S2A). The application of CuO NPs also decreased the concentrations of MMA(V) and DMA(V) in the roots. Similarly, As(III) and As(V) in the rice roots were decreased when the CuO NPs were co-exposed to As(V) (Figure S2B). The CuO NPs that were co-exposed to As(V) also decreased in DMA(V) concentration, while the MMA(V) concentration significantly decreased only under 1B+100 treatment. The addition of 50 and 100 mg L^−1^ CuO NPs significantly reduced the As(III) concentration in the shoots when exposed to As(III) (Figure S2C). The DMA(V) concentration decreased significantly under the 1B+50 and 1B+100 treatments, but there was no significant change in the MMA(V) concentration. Compared to As(V) exposure alone, the addition of CuO NPs significantly decreased the As(III) concentration in the shoots (Figure S2D). The addition of CuO NPs showed no significant difference towards the DMA(V) concentration. The concentration of MMA(V) in the shoots was significantly increased under the 1B+50 treatment, while the 1B+100 treatment significantly decreased the MMA(V) concentration. The concentrations of As(V) significantly decreased under the 0.5B+50 and 1B+100 treatments. Our results show that the addition of CuO NPs reduced the accumulation of different species of As in rice.

### 3.3. Morphology of Rice Root Cell

**Figure 3:**
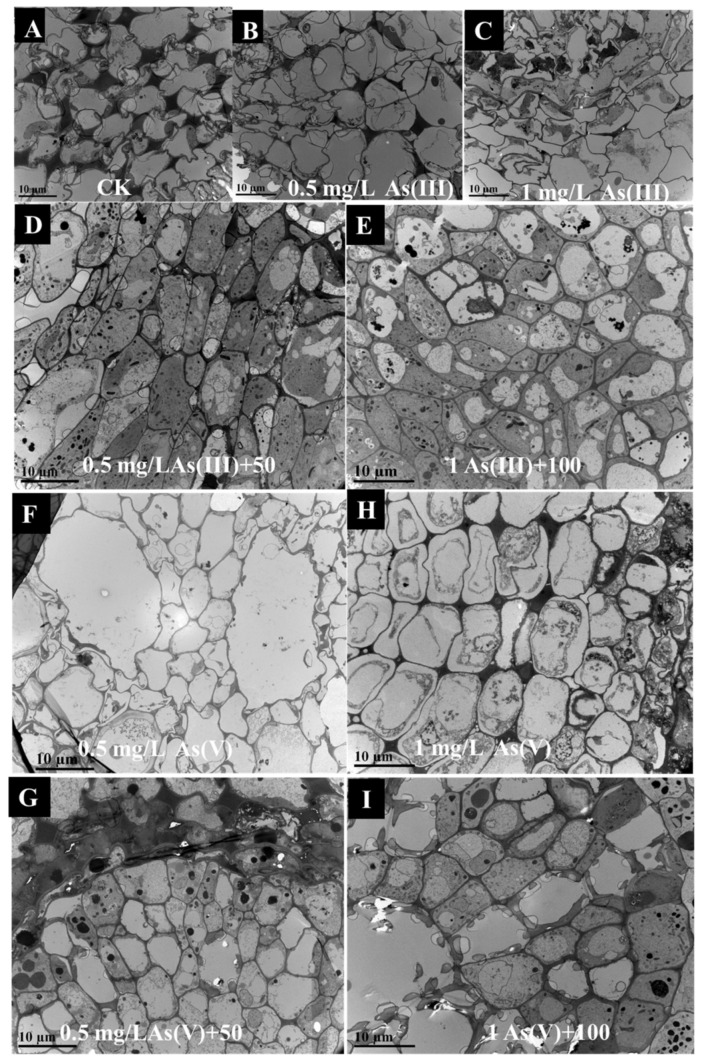
TEM images of root cells under of the CK (**A**), 0.5A (**B**), 0.5A+50 (**C**), 1A (**D**), 1A+100 (**E**), 0.5B (**F**) 0.5B+50 (**G**), 1B (**H**) and 1B+100 (**I**) treatments, respectively.

The morphology of the cells in the roots was observed using TEM. Compared to the CK treatment (Figure 3A), the cell wall of the roots was narrowed under 0.5 and 1 mg/L As(III/V) exposure (Figure 3B,D,F,H). Additionally, the addition of 50 or 100 mg L^−1^ CuO NPs thickened the cell wall of the roots (Figure 3C,E,G,I). The enlarged view of the cells was further observed. The dark substance of high electron density was observed in the epidermal cells and in the intercellular space (Figure S4A–D). Furthermore, EDS was used to analyze the elements of the sediments. The Cu and O were dominant in the substance, especially in the root epidermal cells (Figure S4E). Additionally, the substance in the intercellular space also contained a small amount of Cu (Figure S4F). In addition, the reason for the high content of aluminum (Al) was due to the Al platform that carried the CuO NPs. Our study also found that a slight amount of As was also detected on the dark substance. These results indicate that the CuO NPs accumulated in root epidermal cells and in the intercellular spaces after 14 days of CuO NP exposure.

### 3.4. Relative Expression of As Related Genes in Root

**Figure 4:**
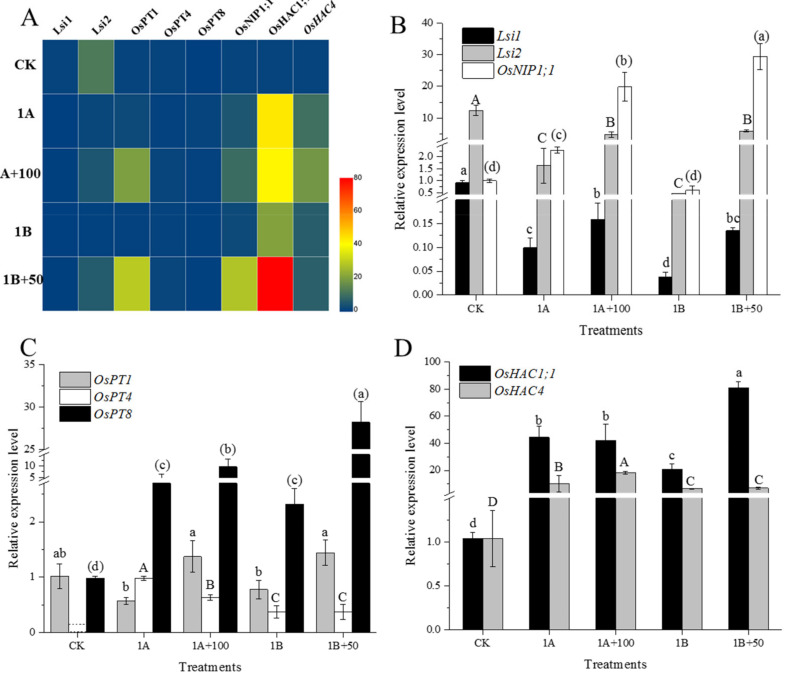
Heatmap (**A**) and relative expression level of As related genes (**B**–**D**) in rice root.

The CK, 1A, 1A+100, 1B, and 1B+50 treatments, which had better rice growth, were selected for qPCR performance in this study. The expression of *OsNIP3;2*, *OsNIP3;3*, and *OsHAC1;2* was not detected in the rice roots. The relative expression level of other the genes involved in As cycling in rice are shown in Figure 4. The heat map shows that the exposure of As or/and CuO NP upregulated the genes for As transport and reduction in root.

The relative expression of different functional genes related to As cycling were further analyzed. The results showed that the As(III) transport and accumulation genes *Lsi1* and *Lsi2* were down regulated after As(III/V) exposure compared to CK treatment (Figure 4B). The relative expression of *OsNIP1;1* was upregulated when exposed to As(III), and As(V) exposure did not significantly change the expression of the *OsNIP1;1* gene. The addition of CuO NPs significantly upregulated the expression of *Lsi1*, *Lsi2*, and *OsNIP1;1* compared to As(III/V) exposure alone. *OsPT1*, *OsPT4*, and *OsPT8* were the genes for As(V) transport and accumulation in rice. The expression of *OsPT1* was down regulated when exposed to As(III/V), while *OsPT8* was upregulated by As, and *OsPT4* was triggered by As(III/V) (Figure 4C). The addition of CuO NPs upregulated the expression of *OsPT1* and *OsPT8* compared to As(III/V) exposure alone. On the contrary, the expression of *OsPT4* was down regulated by CuO NPs and As(III) co-exposure. The addition of CuO NPs did not significantly change the expression of *OsPT4* when As(V) was exposed. The arsenate reduction genes, *OsHAC1;1* and *OsHAC4*, were stimulated by As(III/V) exposure (Figure 4D). There was no significant difference in the expression of *OsHAC1;1* between the As(III) exposure treatment alone and the As(III) co-exposure to the CuO NPs. Moreover, the addition of CuO NPs significantly upregulated the expression of *OsHAC4* when exposed to As(V).

## 4. Discussion

### 4.1. CuO NPs Decreased As(III) Accumulation in Rice

Many studies have shown that CuO NPs has a strong adsorption and oxidation ability for As(III/V) in a wide pH range and in the presence of competitive ions [26,27]. Moreover, As(III) is adsorbed on the surface of CuO NPs and was oxidized to As(V) [28]. There are two possible mechanisms for the oxidation of As(III) to As(V) by CuO NPs: (1) the direct transfer of electrons from As(III) to Cu(II), and (2) the Cu(I) produced on the surface is oxidized back to Cu(II) by dissolved oxygen. Additionally, the second mechanism is the oxidation of As(III) by the reactive oxygen species (ROS) generated by the Cu(I) oxidation process [29]. CuO NPs were separated from the nutrient solution and were analyzed using XPS in our study. Our results showed that As(III/V) was adsorbed on the surface of CuO NPs, and part of As(III) was oxidized to As(V) (Figure 1). In addition, part of the CuO NPs was reduced to Cu(I) under the 1A+100 and 1B+100 treatments. Moreover, As(III) also appeared on the surface of CuO NPs under As(V) treatment in this study, which might be due to the As(III) efflux by rice root and might have increased the As(III) concentration in the solution [30]. The decrease and oxidation of As(III) significantly reduced the accumulation of As(III) in the rice roots and shoots (Figure 2). Therefore, the adsorption of As(III/V) by the CuO NPs was the crucial external factor to reduce As(III/V) accumulation in rice.

The effects of CuO NPs on the morphological changes to the root cell under As stress were further studied. In this study, the cell wall of the rice roots thinned under As stress, while the cell wall thickened after the addition of the CuO NPs. Previous studies have shown that SiO_2_ NPs inhibited As uptake at the single cell level of rice by increasing the cell wall thickness and the mechanical force of the cell wall [31]. Therefore, the thickening of the root cell wall due to the addition of CuO NPs might decrease the transport of As to a certain extent. We also found a large number of CuO NPs in the intercellular space of the root epidermis (Figure S4A–D). The EDS analysis showed that there was only a small amount of As on the surface of the CuO NPs (Figure S4E,F). This result indicates that the CuO NPs had little As adsorption at the single cell level. There were other mechanisms that affected the accumulation and transport of As in rice.

Studies has confirmed that there is an interaction between Cu(II) and As in rice [8], and the accumulation of Cu and As showed a reverse effect when co-exposed to As and the CuO NPs [32]. The same results were also found in our study (Tables S2 and S3, Figure S5). The exposure of the CuO NPs significantly increased the concentrations of Cu, and the As concentrations significantly decreased. There was a negative correlation between the Cu and As concentration in rice (r = −0.182, *p* < 0.05; r = −0.487, *p* < 0.01) when the CuO NPs were co-exposed to As. Therefore, Cu and As might show an antagonistic effect in rice. It is worth noting that higher doses of CuO NPs did not show more of a decrease in the As content in the roots and shoots. Hence, Cu and As might only show an antagonistic effect under lower doses of CuO NPs, and the interaction between Cu and As in rice needs further study. Moreover, the concentration of Cu in roots and shoots sharply increased (Tables S2 and S3), which is toxic to rice growth, and the Cu concentration decreased the biomass (Figure S6). A pot experiment showed that 50 mg kg^−1^ could cause a significant decrease As accumulated in rice and had no inhibitory effect on rice growth [7]. Therefore, lower dosages of CuO NPs could show fewer negative effects on rice growth when the As accumulation in rice is decreased. Our study showed that the phytotoxicity of a high dose of CuO NPs on rice growth would restrict its application in agriculture.

### 4.2. CuO NPs Regulated the Expression of As Related Genes

Studies have shown that Lsi1 is an internal transporter similar to aquaporins, and Lsi2 is an exodus driven by proton gradient; Lsi1 and Lsi2 are located at the distal and proximal ends of the cell membranes in the outer and endodermis and are mainly responsible for the absorption and radial transport of silicon in rice roots [33,34]. Moreover, Lsi1 and Lsi2 were also confirmed to be involved in the absorption and transport of As(III), which mediates As(III) inflow to the cell and outflow to the xylem cells [11,14]. Dose effect was not shown in our study, and of the CK, 1A, 1A+100, 1B, and 1B+50 treatments, which showed higher rice root biomass, were selected to perform qPCR analysis. In this study, the expression of the *Lsi1* and *Lsi2* genes in the rice roots were down regulated when exposed to As(III/V). Therefore, the As(III) accumulation was reduced by the down regulation of *Lsi1* and *Lsi2* expression under As(III/V) exposure. However, the expression of *Lsi1* and *Lsi2* were significantly upregulated after the addition of the CuO NPs in this study (Figure 4B). Additionally, the expression levels of *Lsi1* and *Lsi2* were the highest under the CK treatment, which indicates that *Lsi1* and *Lsi2* were highly expressed in the rice roots when the rice was not subjected to environmental stress. In addition, the adsorption of CuO NPs also decreased the As content in nutrient solution. Therefore, our results showed that the expression level of *Lsi1* and *Lsi2* were regulated by As content in growth medium and were also affected by CuO NPs. Aquaporins OsNIP1:1 provided a way for As(III) to leak from the stele, which limited the transport of As(III) to the xylem and significantly reduced the As(III) transfer from the roots to the shoots and the concentration of As(III) in the shoots [35]. Our results suggested that the expression of *OsNIP1:1* was significantly upregulated when exposed to As(III), while no significant change was observed under As(V) treatment. The *OsNIP1:1* gene was triggered by As(III), and As(V) did not affect the expression of *OsNIP1:1* in this study. Our results also showed that the addition of CuO NPs significantly upregulated the expression of *OsNIP1:1*, which suggests that As(III) transport was restrained by CuO NPs due to the expression of *OsNIP1:1* upregulation. In particular, the expression of *OsNIP1:1* was also upregulated by CuO NPs under As(V) exposure, thereby decreasing the As(III) concentration in the shoots (Figure 2).

The PHT1 family (*OsPT1*-*OsPT13*) consists of the phosphate (Pi) transporter genes in the rice genome and regulate the Pi transporters in the membrane. Previous studies have shown that *OsPT1* is involved in the transport of As(V) from the soil to the apoplast of rice [36]. The *OsPT4* gene is involved in the uptake and transport of As(V) in plants, and the As(V) concentration increased in rice by 23~45% due to the overexpression of *OsPT4* [37]. *OsPT8* has a high affinity for As(V), which is the key transporter of As(V) uptake by rice root. The overexpression of *OsPT8* promoted the uptake of As(V) by rice [38]. Our study found that As(III/V) triggered the expression of *OsPT4* in the roots (Figure 4C). However, the CuO NPs affected the As(V) uptake by down regulating the expression of *OsPT4* under As(III) exposure, and the CuO NPs had no significant effect on *OsPT4* expression under As(V) exposure. Although the addition of CuO NPs upregulated the expression of *OsPT1* and *OsPT8*, the As(V) concentrations in the roots significantly decreased. Previous studies have shown that the decrease of the transporter was responsible for the inhibition of As(V) uptake in rice even though the expression of *OsPT8* was upregulated [39]. Hence, addition of CuO NPs might decrease the As(V) transporter, thereby decreasing As(V) accumulation in rice in this study. In addition, Pi significantly affected the expression of related genes. For example, the expression of *OsPT1*, *OsPT4*, and *OsPT8* were the highest under Pi deficiency, and the expression of *OsPT1*, *OsPT4*, and *OsPT8* in the rice roots were down regulated under As stress [36]. Therefore, the CuO NPs decreased the As content in the growing medium and the increase demand for Pi for plant growth in this study, and the expression of *OsPT1* and *OsPT8* were upregulated by the CuO NPs to uptake more Pi for plant growth.

The arsenate reductase OsHAC1;1 can reduce As(V) to As(III) in rice. The *OsHAC1;1* gene was mainly expressed in the root epidermis, hair, and pericycle. The As(V) induced the overexpression of *OsHAC1;1*, which increased the As(III) efflux, decreasing As accumulation and enhancing the tolerance of the rice seedlings to As(V) [38,40]. Similarly, the As(V) reductase OsHAC4 is induced by As(V) and highly is expressed in the root, which is essential for the reduction of As(V) and As(III) efflux. The expression of *OsHAC4* decreased As accumulation in rice [19]. Our study indicated that CuO NPs regulated the accumulation of As(V) in rice roots by regulating the expression of different genes in this study. CuO NPs promoted As(V) reduction by upregulating the expression of *OsHAC1;1* under As(III) exposure, while the expression of *OsHAC4* was upregulated under As(V) exposure (Figure 4D).

Generally speaking, the decrease of As(III/V) accumulation by CuO NPs in rice was mainly due to the reduction of As(V) and the limitation of As(III) transport. However, the regulation of CuO NPs on the expression of different genes was different under different species of As exposure.

## 5. Conclusions

The application of CuO NPs as fungicides, pesticides and fertilizers, and the accidental leakage of CuO NPs could affect rice growth and metal uptake by rice. A hydroponic culture experiment was conducted to clarify the mechanism of the effect of CuO NPs on the accumulation of different arsenic (As) species in rice. Our results showed that As(III/V) was adsorbed and that As(III) was oxidized on the surface of the CuO NPs. The CuO NPs could prevent As from entering cell by thickening the cell wall. The expression of As(III) transport and the efflux related genes *Lsi1 and Lsi2* were upregulated by the CuO NPs. Additionally, *OsNIP1;1* was upregulated by the CuO NPs, which prevented As transport. The *OsPT1* and *OsPT8* genes involved in As(V) transport were also upregulated by CuO NPs but did not increase As(V) accumulation. When exposed to As(V) or As(III), CuO promoted As(V) reduction by upregulating the expression of *OsHAC1;1* or *OsHAC4*. There was a negative correlation between the concentrations of Cu and As, which indicated that Cu might have had antagonistic effect on As in rice. Our study showed that CuO NPs decreased As(III/V) from efficiently accumulation in rice. Nevertheless, the CuO NPs significantly decreased the rice biomass and increased the Cu accumulation in the rice. In general, the CuO NPs can decrease As accumulation in rice. However, the use of CuO NPs needs to careful attention needs to be paid to avoid yield loss and plant death when using CuO NPs in practical production.

## Data Availability

Data are contained within the article and Supplementary Materials.

## Supplementary Materials

The following are available online at https://www.mdpi.com/article/10.3390/nano11092228/s1, Figure S1: Morphology (A) and elemental composition (B) of CuO NPs, Figure S2: The culture system of rice seedlings in this study. (treatments from left to right in image (A) were CK, 0.5A, 1A, 0.5B and 1B, respectively; (B): 0.5A, 0.5A+50, 0.5A+100, 1A, 1A+50, 1A+100 treatments; (C): 0.5B, 0.5B+50, 0.5B+100, 1B, 1B+50, 1B+100 treatments.), Figure S3: The concentrations and ratios of different species As in the roots (A) and shoots (B), Figure S4: TEM-EDS analysis of rice root under different treatments. D is the magnify view of C. E and F are the EDS spectrum of B and D respectively, Figure S5: Pearson correlation between Cu and As concentration in rice root (A) and shoot (B), Figure S6: Root and shoot biomass of rice, Table S1: Primer sequences for q-PCR, Table S2: The two-way ANOVA and Tukey multiple range tests for the effects of As(III) + CuO NPs on the Cu and As concentrations in rice, Table S3: The two-way ANOVA and Tukey multiple range tests for the effects of As(V) + CuO NPs on the Cu and As concentrations in rice.
