# Supplementary material for: Regulatory Mechanism of Copper Oxide Nanoparticles on Uptake of Different Species of Arsenic in Rice
Source: Nanomaterials (Basel). 2021 Aug 29;11(9):2228. doi: 10.3390/nano11092228 (PMC8469264; doi:10.3390/nano11092228)
Supplement: Supplementary file 1 [file nanomaterials-11-02228-s001.zip › nanomaterials-1333775-supplementary.pdf]

Supplementary Materials

Regulatory Mechanism of Copper Oxide Nanoparticles on Uptake of Different Species of Arsenic in Rice

Qianhua Wu [1,2], Jiyan Shi [1,2,*], Xiaohan Jiang [1,2] and Hanxin Wu [1,2]

1 Department of Environmental Engineering, College of Environmental and Resource Sciences, Zhejiang University, Hangzhou 310058, China; wqhwqh@zju.edu.cn (Q.W.); 11814047@zju.edu.cn (X.J.); wuhanxin@zju.edu.cn (H.W.)
2 Key Laboratory for Water Pollution Control and Environmental Safety, Zhejiang University, Hangzhou 310058, China
* Correspondence: shijiyan@zju.edu.cn; Tel.: +86-0571-88982019

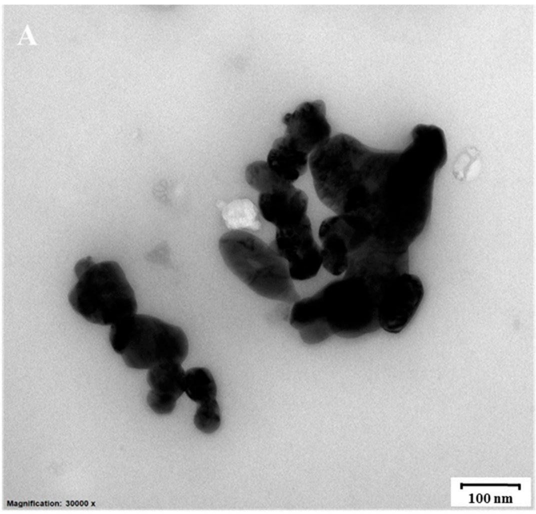

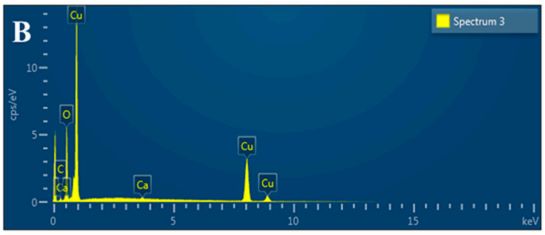

Element	Wt%	Wt% Sigma
C	3.86	0.45
O	21.69	0.30
Ca	0.55	0.11
Cu	73.90	0.45
Total:	100.00	

Figure S1. Morphology (**A**) and elemental composition (**B**) of CuO NPs.

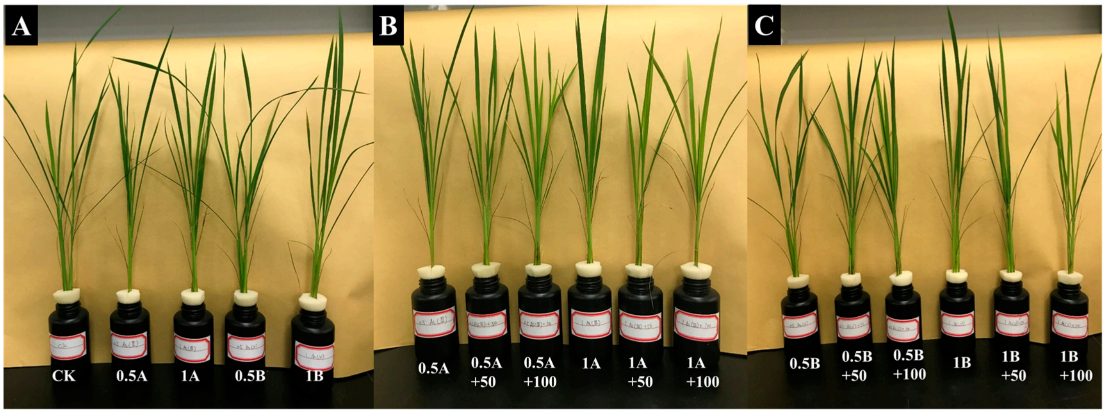

Figure S2. The culture system of rice seedlings in this study. (treatments from left to right in image (**A**) were CK, 0.5A, 1A, 0.5B and 1B, respectively; (**B**): 0.5A, 0.5A+50, 0.5A+100, 1A, 1A+50, 1A+100 treatments; (**C**): 0.5B, 0.5B+50, 0.5B+100, 1B, 1B+50, 1B+100 treatments.).

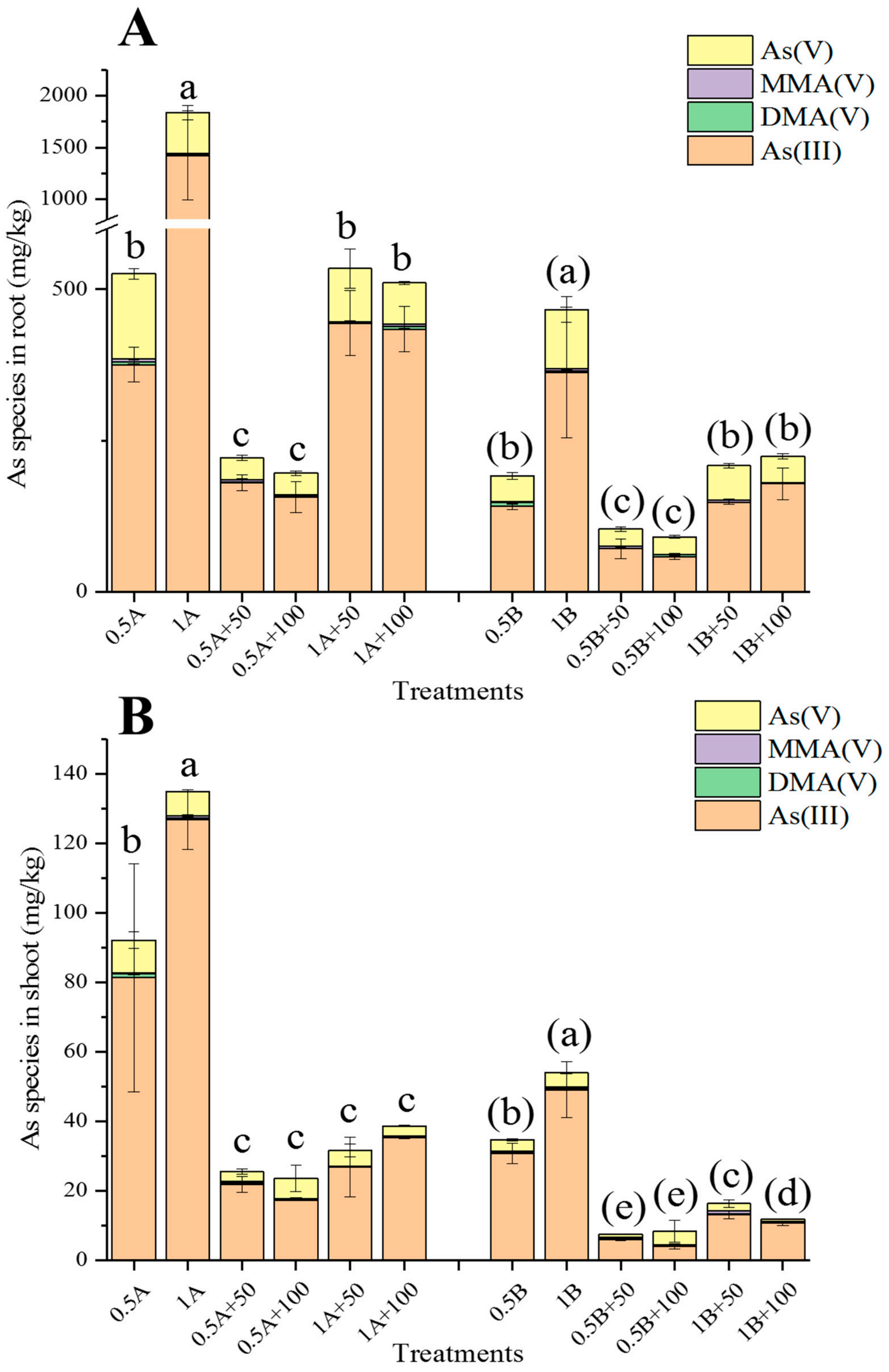

Figure S3. The concentrations and ratios of different species As in the roots (**A**) and shoots (**B**). Different lowercase letters indicate significant differences between different treatments ($p < 0.05$).

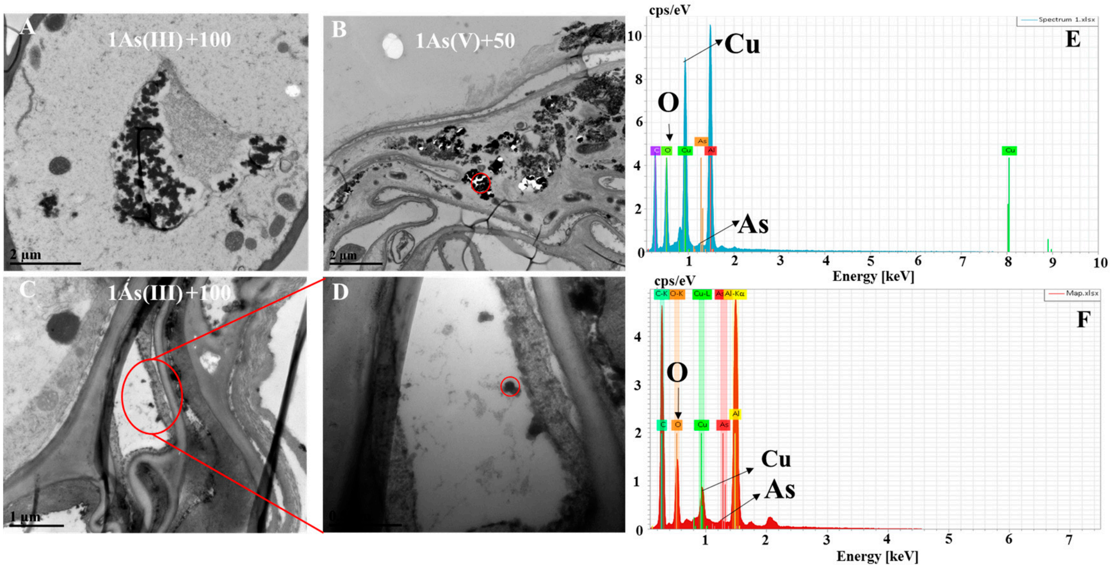

Figure S4. TEM-EDS analysis of rice root under different treatments. **D** is the magnify view of **C**. **E** and **F** are the EDS spectrum of **B** and **D** respectively.

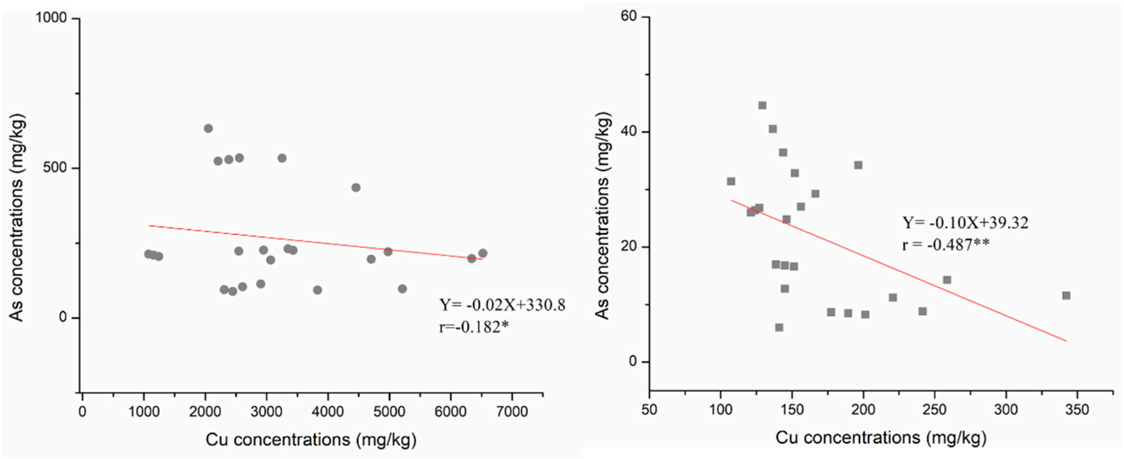

Figure S5. Pearson correlation between Cu and As concentration in rice root (**A**) and shoot (**B**).

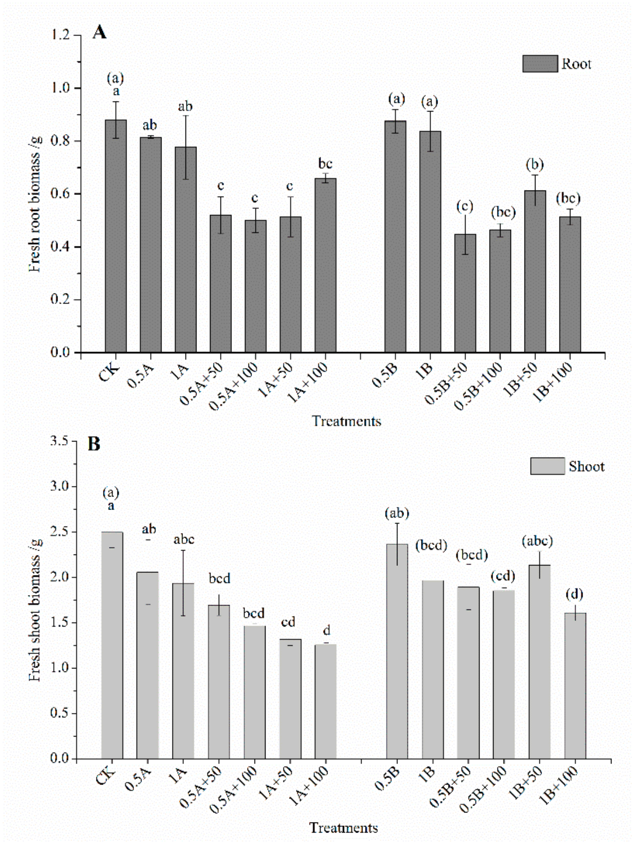

Figure S6. Root and shoot biomass of rice. Different lowercase letters indicate significant differences between different treatments ($p < 0.05$).

Table S1 Primer sequences for q-PCR.

Function	Gene name	Primer	Sequence 5′→3′	References
Transport and efflux of As(III)	*OsNIP3;2*	Former	GTGGCAGTCGGAGCAACAATCAT	Chen et al., 2017
		Reverse	CTTCTCCAGCTACAGCACCAAGTG	
	Lsi1	Former	ATCTACTTCCTGGGCCCAGT	
		Reverse	AGGAGAGCTTCTGGGAGGAG	
	Lsi2	Former	ATCACCTTCCCCAAGTTCC	
		Reverse	CAGCTCCCTCCAGTACATGC	
As accumulatio n	*OsNIP1;1*	Former	GGACTAGTATGGCAGGAGGTGACAACAA	Cui et al., 2020
		Reverse	GGACTAGTTTAGGTGGAGGAGTTCATCC	
	OsNIP3;3	Former	GAAGATCTATGGAAGGGCACAAGAGTGG	
		Reverse	GGACTAGTCTACAGCTTAATTGCAACAT	
Transport of As(V)	*OsPT4*	Former	TTCTGCTAGTGTACCAAACAAAATTACA	Cao et al., 2017
		Reverse	GTAAGTGGCATTTATAATATCAACAGTAA CC	
	OsPT1	Former	CGCTTCCGTACGAGTGGTAGT	Wang et al., 2016
		Reverse	GGTTCTTTCAAATCCAGGGAAA	
	OsPT8	Former	AGAAGGCAAAAGAAATGTGTGTTAAAT	
		Reverse	AAAATGTATTCGTGCCAAATTGCT	
Reduction of As(V)	*OsHAC1;1*	Former	TGAACAAGGGCCATCTACAC	Shi et al., 2016
		Reverse	GACGAGAACTGCTCCACAAA	
	OsHAC1;2	Former	TAGCATCTGCCGATCTCATA	
		Reverse	GAGGTTTATTCACCGCAAGG	
	OsHAC4	Former	TGGCTTCACTCTTCGGCAA	Xu et al., 2017
		Reverse	CATTCTTGAATCCCGCGTCCA	
Actin	*OsActin*	Former	CTTCATAGGAATGGAAGCTGCGGGTA	
		Reverse	CGACCACCTTGATCTTCATGCTGCTA	

References of table S1:

Cao, Y., Sun, D., Ai, H., Mei, H. Y., Liu, X., Sun, S.B., Xu, G.H., Liu, Y.G., Chen, Y.S., Ma, L.N.Q. Knocking Out OsPT4 Gene Decreases Arsenate Uptake by Rice Plants and Inorganic Arsenic Accumulation in Rice Grains. Environ. *Sci. Technol*. **2017**, *51*, 12131–12138.

Chen, Y., Sun, S.K., Tang, Z., Liu, G.D., Moore, K.L., Maathuis, F.J.M., Miller, A.J., McGrath, S.P., Zhao, F.J., 2017. The Nodulin 26-like intrinsic membrane protein OsNIP3,2 is involved in arsenite uptake by lateral roots in rice. *J. Exp. Bot*. **2016**, *68*, 3007–3016.

Cui, J.H., Li, Y.D., Jin, Q., Li, F.B. Silica nanoparticles inhibit arsenic uptake into rice suspension cells via improving pectin synthesis and the mechanical force of the cell wall. *Environ. Sci-Nano*. **2020**, *7*, 162–171.

Shi, S.L., Wang, T., Chen, Z., Tang, Z., Wu, Z.C., Salt, D.E., Chao, D.Y., Zhao, F.J. OsHAC1;1 and OsHAC1;2 Function as Arsenate Reductases and Regulate Arsenic Accumulation. *Plant Physiol*. **2016**, *172*, 1708–1719.

Wang P.T, Zhang W.W, MAO C Z, et al. The role of OsPT8 in arsenate uptake and varietal difference in arsenate tolerance in rice. *Journal of Experimental Botany*, **2016**, *67*: 6051–6059.

Xu, J.M., Shi, S.L., Wang, L., Tang, Z., Lv, T.T., Zhu, X.L., Ding, X.M., Wang, Y.F., Zhao, F.J., Wu, Z.C.. OsHAC4 is critical for arsenate tolerance and regulates arsenic accumulation in rice. *New Phytol*. **2017**, *215*, 1090–1101.

Table S2. The two-way ANOVA and Tukey multiple range tests for the effects of As(III) + CuO NPs on the Cu and As concentrations in rice.

As(III)	CuO NPs	Cu-root	Cu-shoot	As-root	As-shoot
0	0	107.1±4.53 c[(1)]	33.74±0.99 c	ND [(3)]	ND
0.5	0	47.22±19.39 d	76.95±27.46 bc	525.5±41.3 b	105.0±19.5 a
1	0	9.39± 1.12 e	53.37±4.76 c	2112±203 a	136.9±8.12 b
0.5	50	4978±1546 a	124.1±2.91 ab	221.1±4.82 c	26.38±0.40 c
0.5	100	4703±1637 a	156.3±10.12 a	196.0±2.55 c	27.04±3.13 c
1	50	3253±1202 ab	152.0±44.6 a	534.2±98.7 b	32.82±1.41 c
1	100	2383±174 b	136.6±7.24 a	529.1±5.21 b	40.52±5.78 c
Source of variation		Cu-root	Cu-shoot	As-root	As-shoot
As		4.463*[(2)]	2.501	395.5**	219.3**
CuO NPs		31.43**	28.63**	238.5**	238.7**
As*CuO NPs		2.248	2.944	105.8**	13.36**

[(1)] Values are means ± SD (n = 3). Different lowercase letters following values in the same column indicate significant differences ($P \leq 0.05$) between treatments.

([2] F-values for the As(III) treatment and CuO NPs treatment As(III)*CuO NPs interaction. * and ** indicate significant at $P \leq 0.05$ and 0.01, respectively.

[(3)] Values are below detectable limit.

Table S3. The two-way ANOVA and Tukey multiple range tests for the effects of As(V) + CuO NPs on the Cu and As concentrations in rice.

As(V)	CuO NPs	Cu-root	Cu-shoot	As-root	As-shoot
0	0	107.1±4.53 [(1)]d	33.74±0.99 c	ND	ND
0.5	0	80.29±14.18 e	44.21±3.13 c	191.5±34.1 b	38.40±0.69 b
1	0	16.87±0.72 f	37.07±2.14 c	485.5±68.8 a	61.39±0.06 a
0.5	50	2607±298 ab	189.4±12.0 a	104.1±9.44 c	8.47±0.20 e
0.5	100	3830±138 a	241.6±100.6 a	93.02±4.31 c	8.79±2.79 e
1	50	1161±83 c	145.0±6.34 ab	209.6±3.86 b	16.78±0.19 c
1	100	2947±399 ab	208.1±57.9 a	227.0±3.98 b	12.73±1.55 d
Source of variation		Cu-root	Cu-shoot	As-root	As-shoot
As		4.622* [(2)]	0.948	215.2**	1830**
CuO NPs		54.10**	27.28**	75.18**	1911**
As*CuO NPs		2.338	0.282	17.94**	97.52**

(1) Values are means ± SD (n = 3). Different lowercase letters following values in the same column indicate significant differences ($P \leq 0.05$) between treatments.

(2) F-values for the As(V) treatment and CuO NPs treatment As(V)*CuO NPs interaction. * and ** indicate significant at $P \leq 0.05$ and 0.01, respectively.
